# Preparing for cognitive behavioural therapy: a Delphi exercise to develop a consensus curriculum

**DOI:** 10.1136/bmjopen-2024-097681

**Published:** 2025-08-06

**Authors:** Louise Turtle, Nathan Hodson, Helen Wesson, Simon T Williamson, Domenico Giacco

**Affiliations:** 1 University of Warwick, Coventry, UK; 2 Imperial College London, London, UK

**Keywords:** PSYCHIATRY, Adult psychiatry, Anxiety disorders, PUBLIC HEALTH, Depression & mood disorders

## Abstract

**ABSTRACT:**

**Aim:**

To develop a consensus curriculum describing what information service users should be given prior to cognitive behavioural therapy (CBT).

**Design:**

A Delphi study was undertaken. Following development work with professionals and experts by experience, 30 initial statements were prepared and survey rounds were undertaken until 90% of statements achieved 80% consensus on inclusion or exclusion.

**Setting:**

NHS Talking Therapies services in England provide CBT following referral by a professional or self-referral. Out of 1.7 million referrals in 2022–2023 more than 1 million disengaged before or during therapy. Ensuring patients are adequately prepared for CBT is one promising approach to improving engagement and outcomes.

**Participants:**

Participants were only included if they had either 3 years of clinical experience in NHS Talking Therapies services or a leadership role in NHS Talking Therapies services. They reported current employment at seven NHS Trusts in England as well as private practice.

**Results:**

Of the 41 participants, 36 completed all three rounds. After three rounds, 27 statements were included by consensus in the curriculum, covering six domains: Emergency information (two statements), What is CBT? (six statements), Practical preparation for CBT (five statements), What does a CBT session look like? (five statements), What is CBT not? (two statements) and How can people get the most out of CBT? (seven statements).

**Conclusions:**

This consensus curriculum provides a basis for ensuring patients are well-prepared for CBT within the context of NHS Talking Therapies. Further research on improving engagement and outcomes from NHS Talking Therapies services should aim to address these 27 topics.

**Strengths and limitations of this study**

- There was a large and diverse group of participants and we benefited from the input of experts by experience in the development of the study.
- All participants primarily had experience in UK mental health settings, so should be interpreted with caution in psychotherapeutic settings in other healthcare systems.
- Although the cohort was broadly reflective of the British population, this meant that only a small number of included participants were Black and mixed race.

## Background

 The National Institute for Health and Social Care Research recommends cognitive behavioural therapy (CBT) first line for anxiety disorders and for mild and moderate depression.^1 2^ The Improving Access to Psychological Therapies (IAPT) programme, now known as ‘NHS Talking Therapies’, was introduced in 2008 to offer people in the UK with depression or anxiety disorders the opportunity to self-refer for a course of talking therapy typically lasting seven sessions.^3^ The programme produces large reductions in depression and anxiety and a medium-sized improvement in work and social adjustment.^4^

In 2022–2023, 1.7 million people were referred to NHS Talking Therapies. 1.2 million attended the first session, and around 690 000 (40%) completed therapy.^5^ In 2013–2014 there were 1.3 million referrals into IAPT and 37% completed sufficient sessions.^6^ These consistently low completion rates suggest high levels of disengagement and have led to the programme being described as ‘haemorrhaging patients’.^7^ Improving engagement and completion is therefore a key strategic goal for NHS Talking Therapies, and a crucial component of this will be addressing misconceptions about therapy before the first session.^4^ Several national bodies produce patient information leaflets.^8^,^10^ NHS Trusts also often produce materials to help patients know what to expect within their own NHS Talking Therapies teams.^11^ These processes of development and producing information often lead to duplication of effort and producing high quality materials can be very expensive.

Several previous studies have explored interventions designed to increase engagement with psychotherapy, for example questionnaires on personal digital assistants,^12^ online motivational enhancement therapy sessions^13^ or brief informational videos.^14^ However, no previous study has developed a curriculum covering the information service users need before beginning CBT.^15^ In the present study, we use the Delphi method; an iterative approach to reaching expert consensus through repeated rounds of surveys with the same expert participants. The method is well suited to producing consensus curriculums, which reflect a breadth of opinions consolidated through the refinement of key statements.^16^,^19^ Moreover, Delphi studies are an established means to achieve consensus on what information patients should be provided with.^20^ As the current case relates specifically to NHS Talking Therapies, the study was restricted to UK-based participants and may not be extrapolated to other communities.

### Aims

This study aimed to develop a consensus curriculum describing what information service users should be given prior to CBT. The resulting body of accurate, trustworthy and useful information—when given to service users—is intended to better inform prospective patients about what to expect from NHS Talking Therapies and thereby increase attendance.

## Methods

### Patient and public involvement

Patient and public consultation was carried out prior to the main Delphi study and included interviews and a survey to establish if improved information about NHS Talking Therapies would be useful in principle. First, the first and second authors conducted interviews with 31 experts by experience, contacted through NHS Trusts’ patient experience panels. Preliminary interviews were also conducted with charities supporting people living with mental health illnesses. These interviews confirmed a general perception that improved information about CBT and NHS Talking Therapies could better address the concerns of service users, reduce anxiety about beginning treatment and set realistic expectations for therapy.

Second, interviews were conducted with 5 service managers and 15 CBT practitioners or psychotherapists working at NHS Trusts. These interviews were used to generate an initial list of topics for inclusion in the curriculum.

Third, a preliminary survey was produced covering three topics with the potential for inclusion in the curriculum to establish readability. This survey was circulated via NHS Trusts and completed by 41 people, all of whom engaged with the material successfully.

All patient and public consultation was conducted remotely via Microsoft Teams. This patient and public consultation indicated that improved information about NHS Talking Therapies would be a valuable contribution. The insights derived from this consultation were also used to generate the initial draft curriculum.

### Generation of the initial draft curriculum

A steering committee, comprising the study authors, was formed in July 2024. This committee included three psychiatrists with experience of delivering CBT and two project managers with experience of co-ordinating complex data collection tasks. The initial draft curriculum was generated by the first author based on the preliminary patient and public involvement work (including interviews and survey). This draft included a two-level structure with overarching headings covering several specific statements. A second draft was honed by the second and third authors. This draft was reviewed by a therapist who was ineligible for participation in the expert panel and no further changes were proposed. Microsoft Forms was used to host and disseminate the surveys.

### Selection of experts

Experts with past or present experience of delivering NHS Talking Therapies services were initially identified by LT and HAW through personal and professional contacts, with Snowball recruitment used subsequently. Relevant expertise was defined as three or more years of experience as: (a) a psychotherapist (can include training year) or (b) a leadership role within a talking therapies team. Identified experts were initially contacted by email; those who did not meet the above criteria were excluded from the expert panel and did not take part in data collection or analysis. All experts were provided with a participant information leaflet and gave informed consent. Experts were thanked for their participation with a voucher and a certificate. We aimed to include 25–50 expert participants in keeping with other recent Delphi studies.^19^,^22^

### Survey rounds

Survey rounds took place weekly during September and October 2024. In each round, a survey was sent by email to every member of the expert panel. The surveys were titled ‘Before starting CBT, what would it be useful for clients to know?’ and included every proposed statement in the curriculum. Each statement was prefaced with ‘Before starting CBT, it would be useful for my clients to know:’ and followed by a 6-point Likert scale from ‘Strongly Agree’ ‘Moderately Agree’ ‘Somewhat Agree’ to ‘Strongly Disagree’. After rating each statement, participants had the opportunity to suggest a rephrasing or clarification of the statement. An option to suggest additional statements at the end of each topic was also provided. Demographics on participants were collected in the first survey. No translations or plain English version was required. Reminders were sent via email to all participants who had not completed the survey to ensure the rounds progressed at an appropriate rate.

### Analysis

After each round, every statement was anonymised and evaluated for inclusion in the curriculum, exclusion from the study, or further review in the next round.

A statement was included in the curriculum if more than 80% of participants rated the statement ‘Strongly Agree’ or ‘Moderately Agree’, and less than 20% agreed that ‘I would add to this statement/I would change this statement’. Then it was not included in further survey rounds. Although some studies use a definition of consensus below 80%, in this study, we had the capacity to run five full rounds if required, meaning this more stringent requirement was feasible.^16 18 19 23^

If more than 80% of the ratings for a statement were ‘Strongly Agree’ or ‘Moderately Agree’ and 20% or more agreed that ‘I would add to this statement/I would change this statement’ then the statement was reworded based on any feedback and included in the next survey round. A statement was also reworded and included in the next survey round if more than 20% but less than 80% of participants rated it ‘Strongly Agree’ or ‘Moderately Agree’.

Rewording was conducted by the first author by reviewing all the suggested changes and through discussion with the third and fourth authors and no formal qualitative methods were required. Rewording focused on including common suggestions. If there were contradictions, the rewording approach tried to address the majority of concerns. Reworded statements were included in the next survey round.

If less than 20% of the ratings for a statement were 5 ‘Strongly Agree’ or ‘Moderately Agree’ then the statement was excluded from future rounds and was not included in the consensus statement.

### Termination

Survey rounds continued until the termination criteria were met. The termination criteria were met when more than 90% of the statements had been either included or excluded after a minimum of three rounds. At this point, all items which had received consensus were included in the curriculum and all which had not received consensus were excluded from the curriculum. Finally, a survey was circulated to order the curriculum items within topics, with mean scores used to rank statements.

###  Ethical approval

The protocol for this study was submitted to the Biomedical Sciences Research Ethics Committee at the University of Warwick which provided ethical approval (BSREC 15/24–25). The results were reported in keeping with ACCORD statement.^24^

## Results

### Participants

This consensus setting exercise included 41 experts, over three rounds. Participants were predominantly employed by NHS mental health trusts, but one clinical psychologist was currently employed in private practice with previous experience of NHS Talking Therapies. Seven NHS mental health trusts were represented across England, including participants from London, East of England and the West Midlands.

Table 1 describes the characteristics of the participants.

**Table 1:** Participants’ gender, ethnicity, age and role

Demographics	Number of participants	% of participants
Gender		
Female	32	78.05
Male	9	21.95
Ethnicity		
Asian or Asian British	5	12.20
Black, Black British, Caribbean or African	1	2.44
Mixed or multiple ethnic groups	2	4.88
White	31	75.61
Other ethnic group	1	2.44
Prefer not to say	1	2.44
Age range		
18–30	7	17.07
31–40	15	36.59
41–50	10	24.39
50+	9	21.95
Prefer not to say	0	0.00

### Survey rounds

The total number of statements in round one was 30. Round one was completed by 41 participants and 9 (30%) of statements reached the agreement threshold during round one and were added to the consensus curriculum. Based on the participants’ comments, statements which did not reach the agreement threshold were merged, edited and included in round two.

The total number of statements in round two was 18. Round two was completed by 36 participants and 14 (39%) of these statements reached the agreement threshold during round two and were added to the consensus curriculum. One statement met the threshold for exclusion as 93% of participants stated that it should not be included. This statement relates to giving patients information about the training that CBT therapists receive. Based on the participants comments, three statements which did not reach the agreement threshold were edited.

The total number of statements in round three was three. All three of these statements met the agreement criteria and were added to the consensus curriculum. There were no further statements to rephrase. The termination criterion was met on round three and no further rounds were conducted.

### Consensus curriculum

The completed curriculum, broken down by topics and ordered based on expert scores, is presented in table 2. It included six topics covering 27 individual statements. The open dataset is available online.^25^

**Table 2:** A consensus curriculum on preparation for cognitive behavioural therapy (CBT)

Topic 1: Emergency information	
1	It is common for people experiencing low mood or anxiety to have thoughts of self-harm or suicide. Provide links to services and emergency contact numbers.
2	Provide structured activities to create a personal safety plan, with actions like talking to a trusted friend, walking their dog or journaling.
Topic 2: What is CBT?	
3	Thoughts, feelings and actions are connected. Provide definitions of thoughts, feelings (physical sensations and emotions) and actions and examples, along with explanations and examples of the difference between them.
4	CBT helps identify strategies and techniques to promote more balanced and helpful thoughts, feelings and actions and challenge biased or unhelpful ones.
5	Provide the evidence base for CBT, the number of people getting CBT (to normalise it), and the range of problems it treats.
6	Medication and CBT can complement each other, but therapy can be attended without taking medication. It depends on the individual.
7	CBT will not solve all worries or problems, but it will help individuals manage and cope better with them.
8	Provide information about common mental health conditions that CBT can help with: eg, depression, anxiety and Post-traumatic stress disorder (PTSD).
Topic 3: Practical preparation for CBT	
9	Explain how to have a conversation with loved ones about attending therapy, including the potential benefits of CBT for personal relationships and the ways loved ones can support clients.
10	Provide tips for informing and arranging commitments such as work and care commitments.
11	Explain how to prepare for CBT sessions by planning the journey to and from CBT, exploring what a CBT room looks like, or finding a quiet space for online sessions.
12	During the first CBT session, the therapist will get to know the client and understand how they learn best. Clients can openly discuss how therapy fits with their culture, faith or religious practices and request adjustments if needed, such as accommodations for physical or mental disabilities.
13	Suggest bringing a notepad, notebook or digital device to sessions to highlight key learning during or after each session.
Topic 4: What does a CBT session look like?	
14	Between-session work is set collaboratively by the therapist and client. These tasks help implement change outside of sessions, and practising these skills can lead to quicker progress and better outcomes.
15	A CBT session is a partnership between the client and the CBT therapist. The relationship between the CBT therapist and client is important.
16	CBT requires active work and participation.
17	Setting clear goals helps give the client specific aims to work towards. Thinking about what they want to change in their lives and what improvement would look like can help create these goals. Therapists will then help develop these into specific, measurable, acheivable, realistic, and time-limited (SMART) goals.
18	CBT remains valuable even as people improve, allowing them to practise techniques and prepare for challenging times when these skills might be needed.
Topic 5: What CBT is not?	
19	CBT is like other therapies, such as counselling, but also has specific differences. Explanations about these similarities and differences will be provided (to help clients know what to expect and dispel common misunderstandings about CBT).
20	Engaging fully in CBT is important for making changes. Regular attendance is helpful, and frequent cancellations might impact progress.
Topic 6: How can people get the most out of CBT?	
21	It can be helpful to build regular well-being habits, such as improving sleep hygiene, diet or self-reflection, to get the most out of CBT. Content, information and activities to support sleep hygiene, healthy eating, exercise and self-reflection would be provided.
22	CBT helps you learn to self-reflect, so suggest clients start to make reflection a habit beforehand to get the most out of CBT.
23	Suggest clients start reflecting on their goals for CBT in the weeks before they start CBT.
24	Explain how to practise good sleep hygiene.
25	Provide advice on healthy eating (including avoiding alcohol/cigarettes).
26	Recommend regular exercise or time outdoors with variations/reasonable adjustments.
27	Provide advice on relaxation techniques with variations/reasonable adjustments.

## Discussion

### Principle results

Every year, around 1.7 million people are referred for NHS Talking Therapies and more than 1 million drop out before completing the course of therapy.^4^ We aimed to establish a consensus curriculum for information people should know prior to beginning a course of CBT within NHS Talking Therapies services. We conducted a Delphi study including 41 experts from across 7 NHS Trusts plus private practice. Over three survey rounds, there was high engagement with the surveys and the 30 initial statements were edited or merged to generate 27 statements accepted in the consensus statement before the termination criteria were achieved.

### Comparison with the literature

Many interventions have been designed with the aim of promoting engagement with psychotherapies.^15 26^ To our knowledge, this is the first study to systematically develop a consensus curriculum to equip patients for the best outcomes from therapy. One challenge for interventions aimed at improving psychotherapy engagement, noted by Jardine *et al*, is the risk of mismatch between engagement interventions and the stage of change of participants.^15^ The present study has specifically focused on the needs of people who have taken the step of self-referring or seeking referral for CBT, not those who are currently contemplating whether to seek some form of talking therapy. This is not a small target group, encompassing 1.7 million people in the UK each year, but there are nevertheless many people who would benefit from NHS Talking Therapies who have not sought referral and future scholarship should also consider their needs.^5^ Existing interventions to improve engagement with psychotherapy services have taken a diverse range of approaches. Video interventions, web-based programmes and screening questionnaires have all been used.^12^,^14^ Our study fits into this literature by providing a core curriculum of messages which can be delivered through media tailored to the particular target population.

Jardine *et al* noted that one challenge for interventions aimed at improving psychotherapy engagement is the risk of mismatch between engagement interventions and the stage of change.^15^ In the NHS Talking Therapies context, all potential patients have already sought support and many have self-referred.^27^ Therefore, it should be possible to target an intervention giving this information at patients, as they are more likely to be in a similar stage of change.

### Strengths and limitations compared with other studies

This is the first Delphi study to develop a consensus curriculum describing the information patients should know before initiating CBT. Preliminary development work with professionals and experts by experience allowed the team to propose a strong initial curriculum. The large and diverse number of expert participants in the three rounds of the Delphi survey lends credence to the generalisability of the results across different NHS Talking Therapies teams. Other similar studies have generally undertaken two or three rounds and rarely are all five rounds required.^20 22 28^ At 90%, our participant retention was better than other similar studies, possibly because of the offer of vouchers and the momentum maintained by the short gaps between survey rounds.^19^ Although the cohort was broadly reflective of the British population demographics, it may have been informative to have a richer representation of views from Black or mixed race people.

However, caution is required when generalising these results outside of the UK given the particular elements of NHS Talking Therapies CBT services. NHS Talking Therapies services are characterised by waiting lists lasting several months and short courses of as few as six sessions, but free at the point of need. In other contexts, with shorter waiting lists and the expectation of longer lasting courses of therapy, it may be convenient to address many of the items on the curriculum during the first few sessions of therapy. In addition, some patients referred to NHS Talking Therapies are recommended a therapeutic approach other than CBT, in which case some of these recommendations may be less appropriate.

### Implications for policy, practice and research

This consensus curriculum describes the information policymakers should seek to communicate to patients prior to undertaking NHS Talking Therapies. Policymakers at NHS Talking Therapies services should consider means of conveying this information in cheap, scalable and persuasive ways: ‘One drawback of this large curriculum is the risk of non-engagement due to fatigue. Therefore, further research should seek to translate the curriculum into engaging media such as video and audio information.‘’ Practitioners should consider that patients may not be aware of all the information in this curriculum and may wish to share leaflets or websites which can communicate this information with their patients. Researchers should investigate which media most effectively convey this information to patients and evaluate whether the prediction is borne out that better informed patients engage more.^4 26^ Given the significant rise in demand for mental health support in the UK, stopping patients disengaging from NHS Talking Therapies is an urgent policy priority. This consensus curriculum provides a basis for ensuring patients are sufficiently prepared to commit to and benefit from a short course of CBT and would be worthwhile evaluating for its utility in a clinical context.

## Data Availability

Data are available in a public, open access repository.
