# Supplementary material for: Preparing for cognitive behavioural therapy: a Delphi exercise to develop a consensus curriculum
Source: BMJ Open. 2025 Aug 6;15(8):e097681. doi: 10.1136/bmjopen-2024-097681 (PMC12336538; doi:10.1136/bmjopen-2024-097681)
Supplement: Supplementary file 1 [file bmjopen-2024-097681.reviewer_comments.pdf]

PEER REVIEW HISTORY

BMJ Open publishes all reviews undertaken for accepted manuscripts. Reviewers are asked to complete a checklist review form and are provided with free text boxes to elaborate on their assessment. These free text comments are reproduced below.

ARTICLE DETAILS

Title (Provisional)

Preparing for Cognitive Behavioural Therapy: A Delphi Exercise to Develop A Consensus Curriculum

Authors

Turtle, Louise L.; Hodson, Nathan; Wesson, Helen A.; Williamson, Simon T.; Giacco, Domenico

---

VERSION 1 - REVIEW

**Reviewer**	**1**
**Name**	**Jacobsen, Pamela**
**Affiliation**	**University of Bath Department of Psychology, Psychology**
**Date**	**14-Jan-2025**
**COI**	**None**

Thank you for the opportunity to review this paper. It was clearly written and structured throughout. It addresses an area of importance in that drop out rates from NHS Talking Therapy services can be high despite high demand and long waiting lists. I was pleased to read a very practical study with clear implications for clinical practice. The curriculum which is presented in the paper could be used as the gold standard to work from for services wanting to develop patient information literature. A standardised pre-therapy information leaflet for people waiting for CBT via Talking Therapies could also be developed based on this and used across all services nationally.

I have some minor comments which I believe would improve the paper for the authors to consider.

1. The paper states ethics approval came via the university ethics committee, however in several places patients and staff were referred to as being identified and recruited via NHS services, which would require HRA/NHS REC approval. The other alternative would be that the study was given NHS R&D approval as a service evaluation project but as it seemed to cover several NHS trusts this also did not seem viable. Could the authors please clarify this – it could be just that the wording in the paper is unclear and in fact people were not recruited

via NHS services (i.e. via social media/professional networks instead) but its important to address this before the study is ready for publication.

2. In the introduction, the authors make the argument convincingly that no single consensus-based curriculum is available at the moment for CBT preparation. I wonder if it might be helpful to add a couple of sentences acknowledging that patient information is available (a quick google search reveals patient guides freely available on several websites including the NHS, Mind, RCPsych etc.) but these are not necessarily consensus-based and are not standardised. Likewise many individual NHS trusts develop bespoke patient information leaflets but in my experience this often leads to varying quality and a duplication of effort in many cases.

3. In the aims, the term ‘non-adherence’ did not sit quite right with me in the wider context of the paper as it is quite a medicalised term and locates a lot of the power away from the patient who is entitled to make their own decisions about whether a particular treatment is right for them. The problem this study was looking to address is not necessarily that people are not adhering or complying, but that people may find CBT is not quite what they were expecting, which understandably leads them to not continuing with the therapy.

4. The final list of topics to be included in the curriculum all seemed very helpful and relevant, but also very long. There is an obvious tension between comprehensiveness and utility in that a pre-therapy guide which is provided to service users is unlikely to be read and understood if it is very lengthy. In the implications section at the end of the discussion, I would suggest the authors make some comment in relation to this – perhaps acknowledging that further work would be needed to translate the curriculum into a leaflet or video etc. and likely some further prioritisation of key information would be needed.

5. The review copy I had appeared to have a copy of the responses attached as an appendix to the main paper. Not all parts were readable due to truncating of text. It is very helpful to provide an open dataset for reuse or verification of findings, however I would suggest uploading this to a data repository and giving the doi in the paper.

**Reviewer**	**2**
**Name**	**Naeem, Farooq**
**Affiliation**	**University of Toronto Faculty of Medicine, Psychiatry**
**Date**	**23-May-2025**
**COI**	**None**

Dear authors

This is an interesting paper that reports a Delphi exercise on developing a curriculum with the overall aim of improving engagement in NHS talking therapies. I recommend publication with minor revisions to improve the paper.

1 Background last para- please add a few examples. These are mentioned in the discussion, but the background is more appropriate for these

2. The method section is acceptable, but adding details and clarity will help others who want to replicate this methodology. Details of the first qualitative study and the survey will help the researcher understand the context better. You might add the details of these participants, either in the main text or as an appendix. Similarly, the themes derived from this round can be added as an appendix or a table.

My understanding is that the first round of qualitative interviews might have involved in-person or Online meetings, and the second round was conducted through emails. This is not an issue at all; qualitative research offers this flexibility. However, this should be clarified for future researchers who might follow this methodology, especially since consensus conferences are not common. A para on qualitative analyses will also strengthen the paper. Page 6, termination. How many rounds were conducted in total?

3 There is a lot of information at the end of the paper without any headings. Tables and (if there are any) appendices should be referenced in the main body of the manuscript.

4 Table 1. The sample is predominantly white, and while this may reflect this population, it can be mentioned as a limitation considering the diversity of the population in the UK.

5. Discussion. Jardine et al. noted that one challenge for interventions aimed at improving psychotherapy engagement is the risk of mismatch between engagement interventions and the stage of change. You might like to explain how this statement is relevant to your study further. The implication section can also include a real-life evaluation of this curriculum.

Overall, this is a well-conducted study with appropriate methods and descriptions that are an excellent addition to the existing literature on this topic.

VERSION 1 - AUTHOR RESPONSE

Reviewer: 1
Dr. Pamela Jacobsen, University of Bath Department of Psychology
Comments to the Author:
Thank you for the opportunity to review this paper. It was clearly written and structured throughout. It addresses an area of importance in that drop out rates from NHS Talking Therapy services can be high despite high demand and long waiting lists. I was pleased to read a very practical study with clear implications for clinical practice. The curriculum which is presented in the paper could be used as the gold standard to work from for services wanting to develop patient information literature. A standardised pre-

therapy information leaflet for people waiting for CBT via Talking Therapies could also be developed based on this and used across all services nationally.

RESPONSE: Thank you for these kind comments. We do hope this research will be useful to NHS Talking Therapies teams and plan dissemination activities.

COMMENT: I have some minor comments which I believe would improve the paper for the authors to consider.

RESPONSE: Thank you for all these helpful comments which have strengthened the manuscript.

COMMENT: 1. The paper states ethics approval came via the university ethics committee, however in several places patients and staff were referred to as being identified and recruited via NHS services, which would require HRA/NHS REC approval. The other alternative would be that the study was given NHS R&D approval as a service evaluation project but as it seemed to cover several NHS trusts this also did not seem viable. Could the authors please clarify this – it could be just that the wording in the paper is unclear and in fact people were not recruited via NHS services (i.e. via social media/professional networks instead) but its important to address this before the study is ready for publication.

RESPONSE: We only recruited through our professional networks, whether our own or our colleagues' (during the snowball phase). We have amended the manuscript to make this clear.

ABSTRACT "They were drawn from seven NHS Trusts in England as well as private practice" replaced with "Participants were only included if they had either three years of clinical experience in NHS Talking Therapies services or a leadership role in NHS Talking Therapies services. They reported current employment at seven NHS Trusts in England as well as private practice."
PAGE 4 "Firstly, the first and second authors conducted interviews with 31 experts by experience, contacted through NHS trusts." To "Firstly, the first and second authors conducted interviews with 31 experts by experience, contacted through NHS trusts' patient experience panels."
PAGE 5: "Experts were initially identified by LT and HW through contacts at NHS Talking Therapies services" was rephrased as "Experts with experience of NHS Talking Therapies services were initially identified by LT and HW through personal and professional contacts at NHS Talking Therapies services, with Snowball recruitment used subsequently".
"Participants were drawn from NHS mental health trusts, plus one clinical psychologist who currently works in private practice" was replaced with "Participants were predominantly employed by NHS mental health trusts, but one clinical psychologist was currently employed in private practice with previous experience of NHS Talking Therapies."

COMMENT: 2. In the introduction, the authors make the argument convincingly that no

single consensus-based curriculum is available at the moment for CBT preparation. I wonder if it might be helpful to add a couple of sentences acknowledging that patient information is available (a quick google search reveals patient guides freely available on several websites including the NHS, Mind, RCPsych etc.) but these are not necessarily consensus-based and are not standardised. Likewise many individual NHS trusts develop bespoke patient information leaflets but in my experience this often leads to varying quality and a duplication of effort in many cases.

RESPONSE: Thank you for this insightful point. We have borrowed some of this wording to make this important point. "Several national bodies produce patient information leaflets. (CITE) NHS Trusts also often produce materials to help patients know what to expect within their own NHS Talking Therapies teams.(CITE) These processes of development and producing information often lead to duplication of effort and producing high quality materials can be very expensive."

COMMENT: 3. In the aims, the term 'non-adherence' did not sit quite right with me in the wider context of the paper as it is quite a medicalised term and locates a lot of the power away from the patient who is entitled to make their own decisions about whether a particular treatment is right for them. The problem this study was looking to address is not necessarily that people are not adhering or complying, but that people may find CBT is not quite what they were expecting, which understandably leads them to not continuing with the therapy.

RESPONSE: Thank you for drawing our attention to this phrase. We agree that it was not the right phrase. We have replaced that phrase in the aims with: "is intended to better inform prospective patients about what to expect from NHS Talking Therapies and thereby increase attendance."

COMMENT: 4. The final list of topics to be included in the curriculum all seemed very helpful and relevant, but also very long. There is an obvious tension between comprehensiveness and utility in that a pre-therapy guide which is provided to service users is unlikely to be read and understood if it is very lengthy. In the implications section at the end of the discussion, I would suggest the authors make some comment in relation to this – perhaps acknowledging that further work would be needed to translate the curriculum into a leaflet or video etc. and likely some further prioritisation of key information would be needed.

RESPONSE: Thank you for this suggestion. We have now added a sentence on this topic to the implications section: "One drawback of this large curriculum is the risk of non-engagement due to fatigue. Therefore further research should seek to translate the curriculum into engaging media such as video and audio information."

COMMENT: 5. The review copy I had appeared to have a copy of the responses attached as an appendix to the main paper. Not all parts were readable due to truncating of text. It is very helpful to provide an open dataset for reuse or verification of findings, however I would suggest uploading this to a data repository and giving the doi

in the paper.

RESPONSE: Thanks we have added it at the following doi 10.5281/zenodo.15720136 and have removed it from the appendix and cited it in the manuscript: "The open dataset is available from the following DOI: 10.5281/zenodo.15720136."

Reviewer: 2
Dr. Farooq Naeem, University of Toronto Faculty of Medicine
Comments to the Author:
Dear authors

COMMENTS: This is an interesting paper that reports a Delphi exercise on developing a curriculum with the overall aim of improving engagement in NHS talking therapies. I recommend publication with minor revisions to improve the paper.

RESPONSE: Thank you for taking the time to review this manuscript and sharing these helpful comments.

COMMENT: 1 Background last para- please add a few examples. These are mentioned in the discussion, but the background is more appropriate for these

RESPONSE: Thanks we have added the examples in the last paragraph of the background as requested: ", for example questionnaires on personal digital assistants,(12) online motivational enhancement therapy sessions,(13) or brief informational videos.(14)"

COMMENT: 2. The method section is acceptable, but adding details and clarity will help others who want to replicate this methodology. Details of the first qualitative study and the survey will help the researcher understand the context better.

RESPONSE: Thanks for the opportunity to clarify this. These were patient and public involvement activities, so we have now made that clear in the manuscript, and expanded on exactly what they involved.

We have added the title and text: "*Patient and Public Involvement*

Patient and public consultation was carried out prior to the main Delphi study and included interviews and a survey to establish if improved information about NHS Talking Therapies would be useful in principle."

And we have added this explanation: "This patient and public consultation indicated that improved information about NHS Talking Therapies would be a valuable contribution. The insights derived from this consultation was also used to generate the initial draft curriculum."

COMMENT: You might add the details of these participants, either in the main text or as an appendix. Similarly, the themes derived from this round can be added as an appendix or a table.

RESPONSE: Thanks, the themes formed the basis of the initial proposed curriculum for the Delphi study. We have now clearly presented the initial proposed curriculum, which was discussed in round 1 of the Delphi, and explained that it originated from the curriculum work. We did not collect detailed demographic information about the experts by experience who assisted with the patient and public involvement consultation work, beyond the details of their role which is included in the manuscript.

COMMENT: My understanding is that the first round of qualitative interviews might have involved in-person or Online meetings, and the second round was conducted through emails. This is not an issue at all; qualitative research offers this flexibility. However, this should be clarified for future researchers who might follow this methodology, especially since consensus conferences are not common.

RESPONSE: We have now described that all data was collected remotely through surveys and that all preliminary PPI activities involved online interviews. "All patient and public consultation was conducted remotely via Microsoft Teams."

COMMENT: A para on qualitative analyses will also strengthen the paper.

RESPONSE: The PPI work was not formally analysed using qualitative methods. The Delphi research did not involve formal qualitative analysis either. As stated in the submitted manuscript 'no formal qualitative methods were required' for the updating of statements during the Delphi process. We have described the analysis process which was conducted for the Delphi in detail.

COMMENT: Page 6, termination. How many rounds were conducted in total?

RESPONSE: 3 rounds – we have amended the following sentence "The termination criterion was met <u>on round three</u> and no further rounds were conducted."

COMMENT: 3 There is a lot of information at the end of the paper without any headings. Tables and (if there are any) appendices should be referenced in the main body of the manuscript.

RESPONSE: Thanks for this helpful suggestion, these appendices have now been added to the repository and the DOI included in the manuscript.

COMMENT: 4 Table 1. The sample is predominantly white, and while this may reflect this population, it can be mentioned as a limitation considering the diversity of the population in the UK.

RESPONSE: Thank you for this important point which we have added to the 'strengths and limitations' section at the start of the manuscript and in the discussion.

"Although the cohort was broadly reflective of the British population demographics, it may have been informative to have a richer representation of views from Black or mixed race people."

COMMENT: 5. Discussion. Jardine et al. noted that one challenge for interventions aimed at improving psychotherapy engagement is the risk of mismatch between engagement interventions and the stage of change. You might like to explain how this statement is relevant to your study further. The implication section can also include a real-life evaluation of this curriculum.

RESPONSE: Thanks for sharing this interesting insight which we have incorporated into the manuscript. "Jardine et al noted that one challenge for interventions aimed at improving psychotherapy engagement is the risk of mismatch between engagement interventions and the stage of change.(12) In the NHS Talking Therapies context, all potential patients have already sought support and many have self-referred. Therefore, it should be possible to target an intervention giving this information at patients, as they are more likely to be in a similar stage of change."

And added

"and would be worthwhile evaluating for its utility in a clinical context." at the end of the implications section.

COMMENT: Overall, this is a well-conducted study with appropriate methods and descriptions that are an excellent addition to the existing literature on this topic.

RESPONSE: Thank you again for sharing these helpful comments which have strengthened our manuscript.
